# Analysis and design of diode physical limit bandwidth efficient rectification circuit for maximum flat efficiency, wide impedance, and efficiency bandwidths

**DOI:** 10.1038/s41598-021-99405-7

**Published:** 2021-10-07

**Authors:** Babita Gyawali, Samundra K. Thapa, Adel Barakat, Kuniaki Yoshitomi, Ramesh K. Pokharel

**Affiliations:** Graduate School of Information Science and Electrical Engineering, Kyushu University, Nishi-Ku, Fukuoka, 819-0395 Japan

**Keywords:** Energy harvesting, Electrical and electronic engineering

## Abstract

Generally, a conventional voltage doubler circuit possesses a large variation of its input impedance over the bandwidth, which results in limited bandwidth and low RF-dc conversion efficiency. A basic aspect for designing wideband voltage doubler rectifiers is the use of complex matching circuits to achieve decade and octave impedance and RF-dc conversion efficiency bandwidths. Still, the reported techniques till now have been accompanied by a large fluctuation of the RF-dc conversion efficiency over the operating bandwidth. In this paper, we propose a novel rectification circuit with minimal inter-stage matching that consists of a single short-circuit stub and a virtual battery, which contributes negligible losses and overcomes these existing problems. Consequently, the proposed rectifier circuit achieves a diode physical-limit-bandwidth efficient rectification. In other words, the rectification bandwidth, as well as the peak efficiency, are controlled by the length of the stub and the physical limitation of the diodes.

## Introduction

In the wireless power transfer (WPT) and wireless energy harvesting (EH) applications, rectifier always has been an important unit where radio frequency (RF) power must be converted to the direct current (dc) power for powering low power devices like wireless sensor networks, pacemakers, biomedical implants, etc^1–6^. A rectifier with high efficiency is crucial in these fields because the RF-dc conversion efficiency of rectification circuits determines such a system’s overall efficiency. However, high conversion efficiency rectification circuit is achievable only after perfect impedance matching^7,8^ with the source impedance. Furthermore, diode-based rectification circuits pop up with a vast variation in input impedance resulting in impedance mismatching. Therefore, this inappropriate impedance matching has always been accountable to a low efficient rectification circuit.

In the last few decades, high conversion efficiency single-band and dual-band rectifiers for both low power and high power have been investigated and reported^9–17^. Simultaneously, WPT and EH fields are evolved targeting system architectures with multiple frequencies multi-transmitter and receiver, where overall system architecture becomes complex and bulky when multiple narrowband rectifiers are used. Multiband rectifier structures like triple-band, quad-band, six-band have been reported^18–22^ to confront such systems, but they can only handle few specific frequency bands. On the other hand, wideband rectifier circuits can handle a wide range of RF frequencies for DC power conversion. Subsequently, researchers are digging out different topologies to design higher efficiency ultra-wideband rectification circuits.

Recently, several novel techniques to design very low to high power high-efficiency wideband rectifiers for WPT and EH applications have been investigated^23–37^. To cope with the large input impedance variation the design techniques like RF differential two-stage rectifier design^23,32^, complex transmission line broadband matching^24,26–31,33,35–37^, etc., were employed. However, all these works were not only complicated but also came up with complex calculations. In all these works, the wideband power conversion efficiency is fluctuating throughout the frequency range and possesses low impedance bandwidth (IBW) and efficiency bandwidth (EBW). Authors claimed simple wideband rectifier designs had been reported^23–28^, still the conversion efficiency in these designs is limited (typically around 60%), and the efficiency is fluctuating in the working frequency range. Besides, flat efficiency wideband rectifiers have been reported^32,33^, though the EBW is much below 50%. Wideband rectifiers with high IBW and EBW have been reported^29,31,35,36^; however, their circuit size is larger than that of other state-of-the-art. So, wideband rectifiers with high IBW, high EBW, and compact size are complicated to achieve simultaneously. The problem for all these is the difficulty of utilizing the maximum available bandwidth matching for the ultra-wideband rectification.

This paper presents a novel rectification circuit that can utilize the maximum available bandwidth of a conventional voltage doubler circuit for ultrawideband rectification. The proposed novel rectification circuit is realized with a minimal inter-stage matching by a single short-circuit stub and a virtual dc battery. This inter-stage matching self-matches the input impedance of the proposed rectifier circuit to nearly 50 ohms throughout the operating bandwidth. Thus, it eliminates the need for any external matching circuit, which results in extreme circuit miniaturization. Furthermore, maximum efficiency and rectification bandwidth are dependent on the stub length and the diode’s physical limitation. The measurement results show maximum flat conversion efficiency over the entire ultra-wideband operating frequency band, which verifies that the proposed rectification circuit achieved a diode physical limit bandwidth.

## Results

### Theory of the proposed diode physical-limit-bandwidth efficient rectification

**Figure 1:**
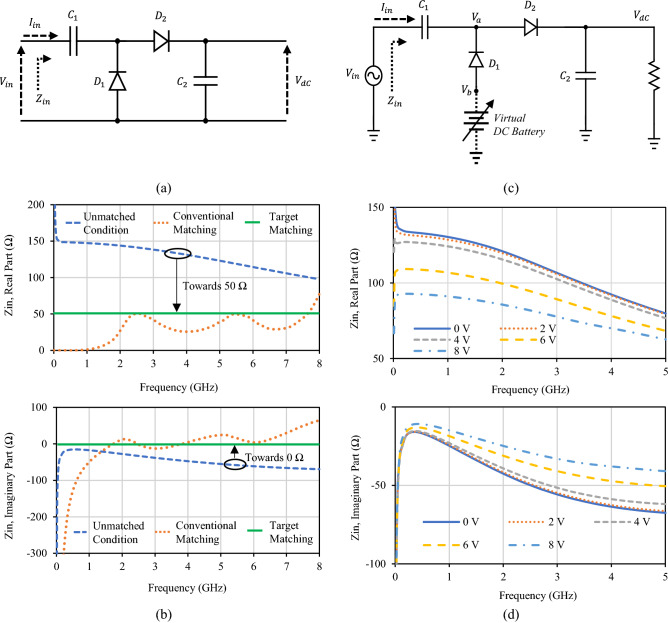
The basic idea of diode physical limit bandwidth rectifier. (**a**) Conventional voltage doubler circuit. (**b)** Comparison of the input impedance of conventional voltage doubler in three different cases. (**c**) Virtual dc battery-based voltage doubler circuit. (**d**) The input impedance of rectifier with varying virtual dc voltage.

The conventional voltage doubler rectifier circuit^7,13,16,21,22,25,26^ is shown in Fig. 1a. The basic components used in this circuit are two Schottky diodes (D_1_ and D_2_), one series pump capacitor (C_1_), and one shunt filter capacitor (C_2_), which is in parallel with the load. The functions of the pump capacitor and shunt filter capacitors are to double the peak output dc voltage and to smooth the output dc voltage by bypassing the higher-order harmonics present in the rectifier output, respectively. Though literature about pump capacitor^38^ and filtering capacitor^39^ estimation are reported, these capacitances should be large enough in voltage doubler circuit to accommodate all the desired frequency band with lower ripple in output voltage. Figure 1b shows the input impedance graph of three cases: unmatched condition, conventional matching, and target matching. The maximum available bandwidth from this voltage doubler circuit is very wide from approximately 0.01 to 5.8 GHz (up to diode operating frequency 5.8 GHz), but the circuit’s input impedance is not 50 Ω, has a non-zero imaginary part, and gradients over the operating frequency band of the rectifier. It is expected to have flat 50 Ω real impedance and flat 0 Ω imaginary impedance over the desired frequency band and by the aid of matching narrowband single- and/or dual-band rectifier circuit can be easily realized as shown with the conventional matching plot in Fig. 1b. A basic requirement for wideband rectification is a lossless varying impedance matching circuit over a wideband frequency range. Indeed, this lossless variable impedance matching circuit is tough to be realized practically. Consequently, the wideband range of this conventional doubler circuit is limited by the designed matching circuit. Therefore, there is a tradeoff in the power conversion efficiency (PCE) of the rectifier for wideband rectifier design with the wide-band operating frequency^23–37^. The idea of lowering the threshold voltage of a diode by increasing temperature^40^ and threshold compensation of transistors^41,42^ have been reported to increase output voltage for energy harvesting techniques. However, diode rectifiers like Schottky Avago HSMS-2862-TR1 diode, the maximum DC voltage across the diode (${V}_{out, DC}$) is limited by the reverse breakdown voltage and the input voltage exceeding this reverse voltage will not have any increment in output DC voltage^43^. Alternatively, we propose a novel concept for achieving efficient wideband rectification without using any external matching circuit as we explain in the succeeding paragraphs.

In the conventional voltage doubler circuit in Fig. 1a, there is not much room to play with the input impedance except the shunt capacitor C_2_ and the load impedance (${R}_{L}$). Instead of direct grounding of diode D_2_ in a conventional voltage doubler circuit, a virtual dc battery was employed to investigate the input impedance of the circuit, as shown in Fig. 1c. The input impedance of the voltage doubler circuit with the virtual dc battery is shown in Fig. 1d, which illustrates that the input impedance can be changed significantly when adjusting the dc voltage of the virtual battery. We explain this behavior by analyzing the circuit at steady-state condition. At the steady-state, D_1_ and D_2_ are always in reverse and forward bias condition, respectively. Then, D_1_ can be represented by its effective junction resistance (${R}_{j}({I}_{b})$) and capacitance (${C}_{j}$(${V}_{ba}$)), which are calculated by (1) and (2), respectively.

1 $${R}_{j}\left({I}_{b}\right)= \frac{8.33\times {10}^{-5}\times N\times T}{{I}_{b}+{I}_{s}},$$

2 $${C}_{j}\left({V}_{ba}\right)\hspace{0.17em}={C}_{j0}{\left(1-\frac{{V}_{ba}}{{V}_{\mathrm{\O }}}\right)}^{-M},$$

where ${I}_{b}$ is external bias current in μA, ${I}_{s}$ is saturation current in μA, $N$ is identity factor, $T$ is the temperature in ˚K and ${V}_{ba}$ is the dc voltage across the junction, ${C}_{j0}$ is the junction capacitance value when ${V}_{ba}$ = 0, $M$ is the junction potential gradient, and ${V}_{\mathrm{\O }}$ is junction potential. The introduction of the virtual battery, ${V}_{b}$, reduces ${V}_{ba}$ leading to an increased capacitance ${C}_{j}$(${V}_{ba}$). Also, ${I}_{b}$ increases leading to reduced ${R}_{j}({I}_{b})$. These two phenomena explain the reasons behind the changes in input impedance when a virtual dc battery exists.

This shows that we can achieve some self-matching of voltage doubler circuit by using virtual dc battery for reverse biasing on shunt diode D_1_ instead of direct grounding. In the actual application, there is no point in using a battery to design a rectifier circuit. Therefore, we propose an alternative way to generate a dc voltage supply to shunt diode D_2_ to achieve this advantage of a virtual dc battery for impedance matching on the conventional voltage doubler circuit for wideband rectifier designs.

### Proposed rectifier and virtual battery realization

**Figure 2:**
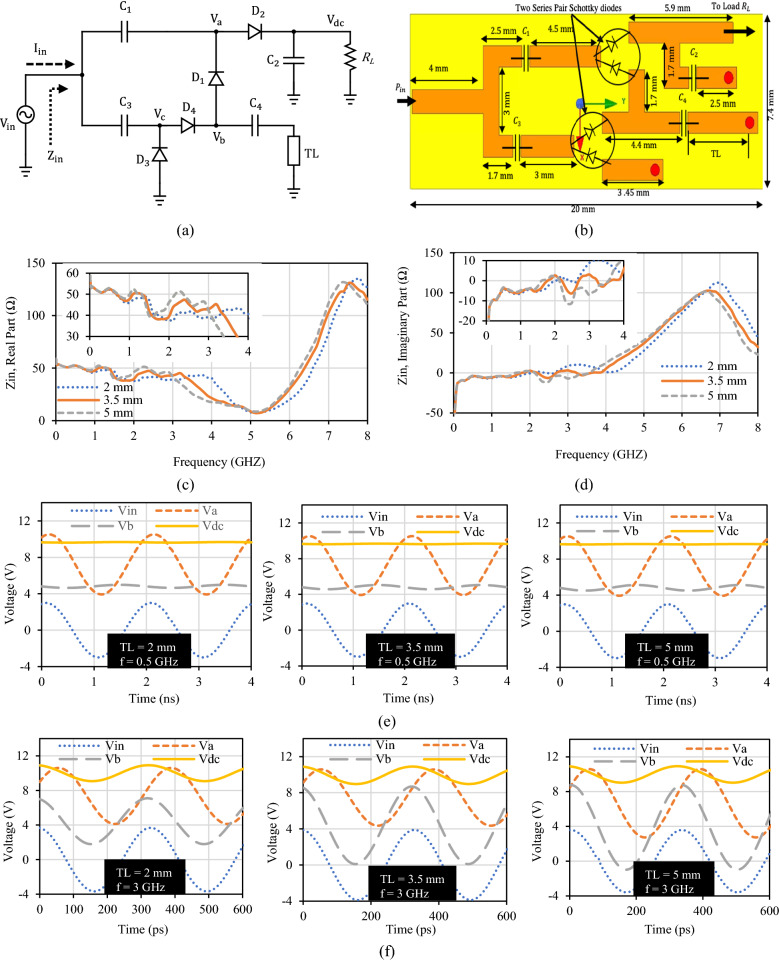
Proposed diode physical limit bandwidth rectifier. (**a**) Schematic. (**b**) Layout mask. *C*1 = *C*2 = *C*3 = *C*4 = 100 pF, *TL* = 3.5 mm*.* (**c**) Real input impedance of proposed rectifier with varying stub length. (**d**) Imaginary input impedance of proposed rectifier with varying stub length. (**e**) Voltage waveforms of the proposed rectifier circuit at 0.5 GHz for different stub lengths. (**f**) Voltage waveforms of the proposed rectifier circuit at 3 GHz for different stub lengths.

A rectifier cannot have an actual dc source or battery. So, instead, we can implement a virtual battery. This virtual battery can be formed by a secondary voltage doubler (consisting of capacitors C_3_ and C_4_, diode D_3_ and D_4_) that share the same RF input signal as shown in Fig. 2a. The capacitor C_3_ is selected the same as C_1_ and C_2_ for simplicity. The final layout mask of the proposed rectifier circuit is shown in Fig. 2b. Electromagnetic (EM) simulation is done for transmission lines in this layout. After the EM simulation of transmission lines layout along with the capacitors and diodes spacing in the High Frequency Structure Simulator (HFSS), a multiport S-Parameter file was generated. Since diodes cannot be handled in HFSS 3D EM simulation software, later, this multiport S-Parameter from HFSS was imported to Keysight ADS as a data item for further analysis with diodes existing. When the overall circuit impedance of the proposed rectifier circuit is observed with both schematic and layout generated from EM simulations, it was found that this virtual dc voltage realization by additional stage of voltage doubler circuit pulls down the real part of circuit input impedance to almost 50 Ω and pulls up the imaginary part of circuit input impedance to nearly 0 Ω as presented in Fig. 2c,d. This is because the input impedance is the function of two parallel voltage doubler circuits, and the total impedance becomes halved.

As shown in Fig. 2a,b, a short stub transmission line (TL) is used to terminate the capacitor C_4_ instead of direct grounding. C_4_ is a dc block capacitor, which is an open circuit at dc. At 320 MHz, C_4_ together with the stub transmission line realize an ac ground. To utilize the physical limit of the diode, we have designed this transmission line length such that the overall circuit produces parallel resonance around 7 GHz. Hence, at the bandwidth of interest two targeted design issues are achieved within the physical-limit bandwidth, which are (i) The real part of the impedance is flattened to 50 Ω. (ii) The imaginary part of the impedance is pulled to zero. When varying the stub length, the effect of this resonance on the real and imaginary parts of the input impedance of the proposed circuit is as shown in Fig. 2c,d, respectively. When the input impedance was observed at TL = 5 mm, although this stub length provides almost perfect 50 Ω input impedance but the impedance bandwidth is only up to 2.9 GHz. We can further decrease the length of this stub to achieve an ultra-wideband response. With the stub length, TL = 2 mm, larger bandwidth up to 4.3 GHz is achievable but both the real and imaginary part input impedance deviates much from 50 Ω matching impedance. When the stub length is minimal, we can get extra wideband as compared to the long stub length but at the same time, we need to compromise with impedance matching. So, to obtain maximum flat efficiency in the entire desired wideband from this proposed rectifier, a precise selection of the short stub is necessary. Therefore, the stub length of TL = 3.5 mm was selected after the optimization, which possesses both non-fluctuating 50 Ω input impedance and larger bandwidth with minimal effect on the conversion efficiency and the output voltage.

Here we discuss about the stub to illustrate its function. The voltage waveforms at different positions of the proposed voltage doubler circuit while varying the stub length at 0.5 GHz and 3 GHz are shown in Fig. 2e,f, respectively. At a stub length TL = 2 mm, perfect dc voltage appears at point ${V}_{b}$ when the frequency is 0.5 GHz. But when the frequency is 3 GHz, ${V}_{b}$ fluctuates and the rectifier starts to show more ac response at this point. Similar effect appears for other stub-lengths. However, the longer the stub length, the more ${V}_{b}$ fluctuations amplitude. Even, at 3 GHz, a negative ${V}_{b}$ value start appearing when the stub length is 5 mm. Therefore, the stub length (TL) selection should be made comprehensively to achieve dc output voltage at both lower and higher frequencies. Thus, in our proposed wideband rectifier design, we have selected 3.5 mm length for the stub to achieve best matching as well as best dc voltage over the operating ultrawideband frequency.

### Fabrication and measurement results

**Figure 3:**
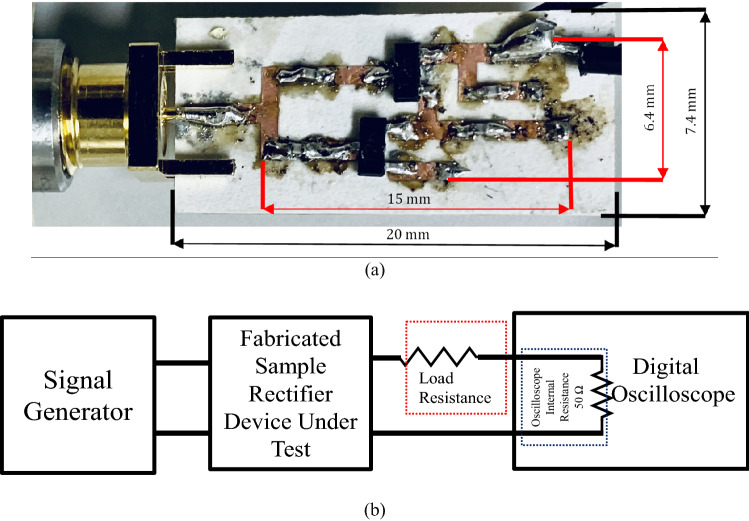
Fabrication and measurement setup of the proposed diode physical-limit bandwidth rectifier circuit. (**a**) Fabricated sample photograph. (**b**) Measurement setup.

A sample of the proposed diode physical-limit bandwidth rectifier was fabricated using Rogers3003™ substrate. Figure 3 shows the fabricated sample photograph and measurement setup of the proposed diode physical-limit bandwidth rectifier circuit. Then, the performance of the rectifier was verified by measuring the input reflection coefficient and the efficiency calculated from the measured output DC voltage. Measurement data were taken for a wide range of frequency, input power, and load to obtain sufficient demonstration.

**Figure 4:**
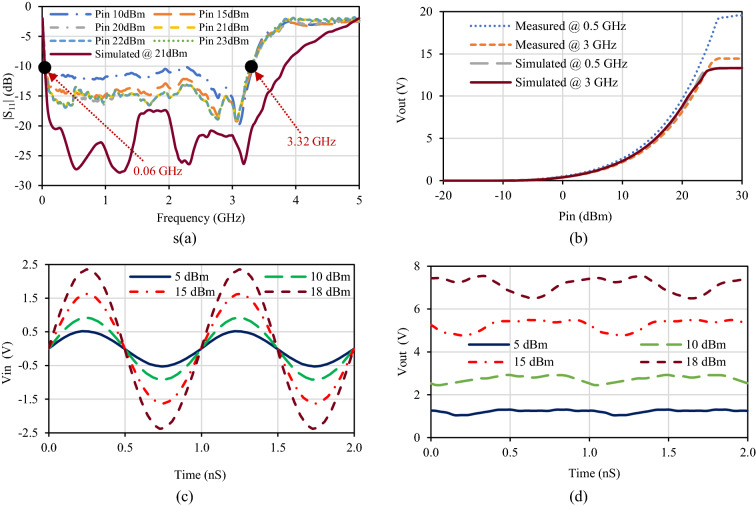
Proposed diode physical-limit bandwidth rectifier’s simulated and measured results. (**a**) Input reflection coefficient |S_11_| at different input power levels. (**b**) Output voltage versus input power level at different frequencies. (**c**) Oscilloscope measured input voltage waveform for different input power levels at 1 GHz. (**d**) Scaled oscilloscope measured output voltage waveform for different input power levels at 1 GHz and 1.3 KΩ.

The reflection coefficient (|S_11_|) of the fabricated rectifier was observed using the PNA Network Analyzer. Figure 4a shows the simulation and the measurement result of the input reflection coefficient of the fabricated rectifier at different input power levels at optimal efficiency loads. Although there is a slight change in the reflection coefficient bandwidth, the measurement result well captures the tendency of the input reflection coefficient of simulation. This change in the reflection coefficient is most possibly due to the package parasitic capacitance effect, which was not considered during simulations. From the measured reflection coefficient, it is seen that the rectifier circuit provides a reflection coefficient of less than − 10 dB over the frequency range from 0.06 to 3.32 GHz, which corresponds to a calculated IBW of 192.9% using Eq. (3). Moreover, this IBW is valid for a wide range of input power levels from 10 to 27 dBm.

3 $$IBW= \frac{{f}_{u, |S11|}-{f}_{l, |S11|}}{\left(\frac{{f}_{u, |S11|}+{f}_{l, |S11|}}{2}\right)} \times 100\%,$$

where, ${f}_{l, |S11|}$ and ${f}_{u, |S11|}$ are lower and upper frequencies, respectively, indicating − 10 dB bandwidth on reflection coefficient of |S_11_|.

Figure 4b shows the simulated and measured output voltage of the fabricated rectifier. In Fig. 4c,d oscilloscope measured input and output voltage waveforms are shown for four different input powers (Pin = 5 dBm, 10 dBm, 15 dBm, and 18 dBm). The measurement maximum input power was limited by the used Tektronix digital phosphor oscilloscope (Part #DPO 70404C) measurement limit. The oscilloscope measured voltage is recorded as the voltage drop within oscilloscope internal resistance 50 Ω as shown in the measurement setup in Fig. 3b. Then, output voltage plotted in Fig. 4d was calculated as,

4 $${V}_{out}=\left(\frac{{R}_{L}}{50}+1\right)\times {V}_{osciliscope}.$$

The measured output voltage at the instant Pin = 15 dBm at 1 GHz input power is almost exact and the oscilloscope measured output dc voltage is almost flat as can be interpreted from Fig. 4d.

The conversion efficiency is the ratio of output DC power delivered to the load impedance ${R}_{L}$ to the power delivered by the source at the input of the rectifier circuit, which is represented by,

5 $$\upeta =\frac{{P}_{out}}{{P}_{in}}\times 100\mathrm{\% }=\frac{{{V}_{DC}}^{2}}{{R}_{L}{P}_{in}}\times 100\mathrm{\%}.$$

**Figure 5:**
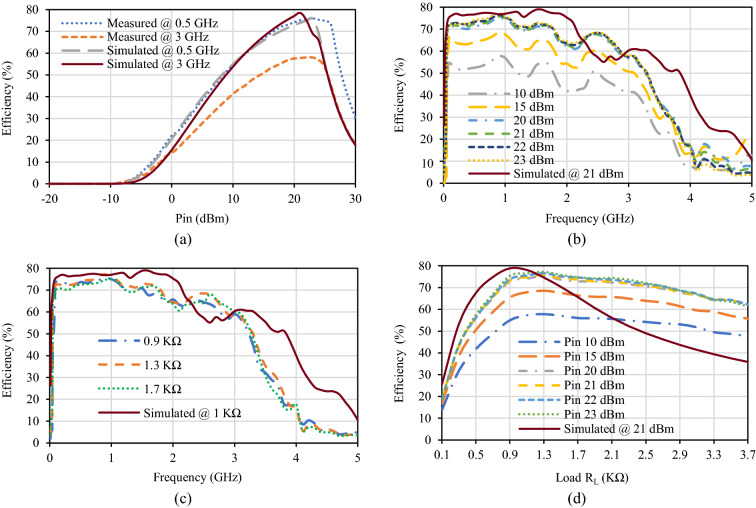
Proposed diode physical-limit bandwidth rectifier’s simulated and measured results. (**a**) Efficiency versus input power at different frequencies. (**b**) Efficiency versus frequency at different input power levels. (**c**) Efficiency versus frequency at different loads. (**d**) Efficiency versus load at different input power levels.

The rectifier conversion efficiency is computed and plotted in Fig. 5 in various conditions. The simulated maximum conversion efficiency obtained is 78.867% at 21 dBm input power with a load impedance of 1 KΩ at 1.5 GHz frequency whereas the measured maximum conversion efficiency obtained is 77.3% at 23 dBm input power with a load impedance of 1.3 KΩ at 0.9 GHz frequency. The conversion efficiency over the operating frequency bandwidth with different input power levels at optimal efficiency loads is shown in Fig. 5b. The efficiency remains above 50% throughout the entire IBW from 0.06 to 3.32 GHz at input power levels from 10 to 27 dBm. For the input power of 20 dBm to 23 dBm, the conversion efficiency remains above 70% at the frequency range 0.06 to 1.82 GHz. The calculated efficiency bandwidth (EBW) using Eq. (6) is 187.23% (maintaining conversion efficiency > 70%) whereas 192.9% (maintaining conversion efficiency > 50%), which is the highest ever recorded than other reported wideband rectifiers^23–37^. Moreover, the efficiency over the entire operating bandwidth has negligible fluctuations.

6 $$EBW= \frac{{f}_{u, \eta }-{f}_{l, \eta }}{\left(\frac{{f}_{u, \eta }+{f}_{l, \eta }}{2}\right)} \times 100\%,$$

where, ${f}_{l,\upeta }$ and ${f}_{u,\upeta }$ are lower and upper frequency indicating efficiency bandwidth on efficiency vs. frequency graph for efficiency greater or equal to the specified percentage.

The simulated and measured conversion efficiencies at different loads with optimal efficiency input power over the operating frequency bandwidth is presented in Fig. 5c, whereas over the varying load at different input power levels at optimal efficiency frequency is presented in Fig. 5d. From these graphs, it can be interpreted that the proposed rectifier supports a wide range of loads while maintaining maximum flat efficiency.

A performance comparison of our proposed diode physical-limit bandwidth rectifier with other reported wideband rectifiers is summarized in Table 1. It can be interpreted that the proposed diode physical-limit rectifier bandwidth has the highest ever achieved IBW of 192.9%, the highest ever achieved EBW of 192.9%, and the minimal circuit size while maintaining the flat rectification efficiency of above 50% from 0.06 to 3.32 GHz for input power levels from 10 to 27 dBm, and above 70% from 0.06 GHz to 1.82 GHz for input power levels from 20 to 23 dBm. Moreover, this proposed diode physical-limit bandwidth rectifier presents a peak rectification efficiency of 77.3% at the input power level of 23 dBm, the load impedance of 1.3 KΩ and frequency of 0.9 GHz.

**Table 1:** Rectifier performance comparison with other related state-of-the-art.

References	Frequency range [GHz]	Conversion efficiency (%)	IBW (%)	EBW (%)	Peak efficiency (%)	Area (cm^2^) {active area}
^26^	1.65–3.05	$\ge$ 50	33.2	57 @ 15 dBm	78.3 @ 18 dBm	24.8
^29^	0.54–1.3	$\ge$ 50	76	82.6 @ 5 dBm	80 @ 10 dBm	6.7
^30^	2–3.05	$\ge$ 70	50	41.5 @ 10 dBm	75.8 @ 14 dBm	12.6
^31^	0.6–3	$\ge$ 50	85.7**	133.3 @ 17 dBm	66 @ 17 dBm	3.25
^32^	2.08–2.58	$\ge$ 70	N.A.	21.5 @ 10 to 18.6 dBm	80.8 @ 17.2 dBm	N.A.
^33^	0.57–0.90	$\ge$ 70	48.3	44.9 @ 12.8 dBm	75** @ 14.1 dBm	46.4
^36^	1.4–3.7	$\ge$ 50	71.6	90.2 @ 10 dBm	75** @ 10 dBm	8.75
^37^	0.78–1.43	$\ge$ 50	N.A.	58.8 @ 14 dBm	81 @ 14 dBm	N.A.
This work	0.06–3.32	$\ge$ 50	192.9	192.9 @ 10 to 27 dBm	77.3 @ 23 dBm	1.48
	0.06–1.82	$\ge 7$0		187.2 @ 20 to 23 dBm		{0.96}

IBW and EBW were calculated with center frequency of operating bandwidth.

*N.A.* information not provided.

**Is extracted data from the graphs of corresponding references.

## Conclusion

In this paper, we presented a diode physical-limit-bandwidth efficient rectification circuit. This novel rectification circuit was achieved with minimal inter-stage matching consists of a single short-circuit stub and a virtual battery, which contributes negligible losses. Such a circuit eliminated the need for complex matching elements to realize octave or decade impedance in conventional voltage doubler circuits. Rectification bandwidth and maximum flat conversion efficiency can be controlled by the length of the interstage stub and the physical limitations of the used diodes. This proposed rectification circuit was fabricated and measured for verification. Measurement results of this novel rectification circuit were in good agreement with the simulation results. Finally, the presented results showed that this proposed novel rectification circuit achieved maximum flat efficiency over the entire ultra-wide rectification bandwidth from 0.06 to 3.32 GHz and outperforms other reported state-of-arts. Therefore, this ultra-wideband rectification circuit can be considered as the best candidate for compact size high-efficiency wideband wireless EH and WPT system applications.

## Methods

### Circuit and electromagnetic simulations

We used Keysight Advanced Design System software (ADS version #2014.01) for simulation and optimization of rectifier circuit layout dimensions, and ANSYS High-Frequency Structure Simulator (HFSS version #2018.01) for electromagnetic simulations of the final layout.

### Fabrication of samples

We fabricated the prototype using the MITS FP-21T Precision milling machine.

### Materials

We have used Rogers RO3003 (dielectric constant = 3, height = 0.762 mm, and copper thickness = 17 µm) during both simulations and the prototype’s preparation. Lumped capacitors used were high-quality factor GJM series capacitors from Murata electronics. Schottky Avago HSMS-2862-TR1 diode model^44^ (*B*_*v*_ = 7.0*V,* *V*_*th*_ = (0.25–0.35)*V, R*_*s*_ = 5$\Omega$ *C*_*j0*_ = 0.18 pF*, I*_*s*_ = 5 × 10^−8^ *A, M* = 0.5*, N* = 1.08) have been used as the rectifying diode to get the maximum rectification efficiency in wideband operating frequency. The reason to choose this diode is that it possesses low threshold voltage (Vth), low non-linear resistance (Rs), and high breakdown voltage (${B}_{v}$) to achieve maximum efficiency in the ultrawide operating bandwidth for WPT applications.

### Measurement setup: for reflection coefficient

The measurement setup to measure the reflection coefficient of the fabricated rectifier circuit consists of Keysight PNA series vector network analyzer (Part #N5222A), radiofrequency cables, resistors, and breadboard. Signals of different power and frequencies were subjected from PNA through a coaxial cable to the rectifier prototype, and the reflection coefficient was recorded for different resistive loads conditions. The measured reflection coefficient result for optimal efficiency load resistance is shown in Fig. 4a for different input power levels.

### Measurement setup: for efficiency calculation

The measurement setup to compute the rectification efficiency of the proposed rectifier consists of mainly an Anritsu vector signal generator (Part #MG3710A), radiofrequency cables, digital multimeter, resistors, and breadboard. The RF power supply for the rectifier is provided from a vector signal generator through a coaxial cable. The dc output voltage is measured using a digital multimeter. The input power level was first set at − 20 dBm, and with the increasing steps of 1 dBm up to 30 dBm, the output voltage was recorded. Moreover, different input powers with different frequencies were delivered from the signal generator to the rectifier circuit for different loads, and the output voltage was measured. Later, rectification efficiency was computed from this measured voltage under various conditions.

### Measurement setup: for input–output voltage waveform

A schematic of the measurement setup to measure the input and output voltage waveform consists of an Anritsu vector signal generator (Part #MG3710A), Tektronix digital phosphor oscilloscope (Part #DPO 70404C), radiofrequency cables, resistors, and breadboard. Input and output voltages were recorded from an oscilloscope.
