# Author Correction: Analysis and design of diode physical limit bandwidth efficient rectification circuit for maximum flat efficiency, wide impedance, and efficiency bandwidths

**DOI:** 10.1038/s41598-022-09087-y

**Published:** 2022-03-22

**Authors:** Babita Gyawali, Samundra K. Thapa, Adel Barakat, Kuniaki Yoshitomi, Ramesh K. Pokharel

**Affiliations:** Graduate School of Information Science and Electrical Engineering, Kyushu University, Nishi‑Ku, Fukuoka, 819‑0395 Japan

Correction to: *Scientific Reports* https://doi.org/10.1038/s41598-021-99405-7, published online 07 October 2021

The original version of this Article contained an error in the Acknowledgements section.

“This work was supported in part by JSPS KAKENHI Grant Number: 21K04178, in part by the MIC/SCOPE Grant Number: 21452069, in part by the Foundation for Technology Promotion for Electronic Circuit Board, and in part by a research grant from The Murata Science Foundation.”

now reads:

“This work was supported in part by the MIC/SCOPE Grant Number: JP215010003, in part by JSPS KAKENHI Grant Number: 21K04178, in part by the Foundation for Technology Promotion for Electronic Circuit Board, and in part by a research grant from The Murata Science Foundation.”

The original Article has been corrected.

